# Identification of Key Regulators Mediating Gamma-Aminobutyric Acid (GABA) and Organic Acid Accumulation in Strawberry

**DOI:** 10.3390/plants14223437

**Published:** 2025-11-10

**Authors:** Lingzhi Wei, Shuangtao Li, Rui Sun, Yongqing Wei, Hongli Zhang, Linlin Chang, Chuanfei Zhong, Jing Dong, Guixia Wang, Jian Sun

**Affiliations:** 1 Institute of Forestry and Pomology, Beijing Academy of Agriculture and Forestry Sciences, Beijing 100093, China; 2 Key Laboratory of Biology and Genetic Improvement of Horticultural Crops (North China), Ministry of Agriculture and Rural Affairs, Beijing 100093, China; 3 Beijing Engineering Research Center for Strawberry, Beijing 100093, China

**Keywords:** strawberry, antioxidants, γ-aminobutyric acid (GABA), organic acids, glutamate decarboxylase (*GAD*)

## Abstract

Growing market demand exists for strawberry, with nutrient-rich and health-promoting properties, beyond mere taste and flavor. Genetic biofortification is a powerful strategy to enhance nutrient metabolites in strawberry. Both GABA and organic acids contribute to human health by supporting nervous system relaxation and enhancing metabolic and digestive functions, respectively. However, the regulatory mechanisms underlying their accumulation remain poorly understood. In this study, we analyzed the accumulation patterns of GABA and organic acids across four fruit developmental stages in two representative cultivars, ‘Monterey’ and ‘Benihoppe’. Ripening ‘Benihoppe’ fruits accumulated higher levels of GABA and citric acid, whereas ‘Monterey’ fruits contained more malic acid. Integrated transcriptome analysis identified key structural genes and transcription factors (TFs) involved in GABA biosynthesis. Notably, functional characterization revealed that *FaGAD4* significantly promotes GABA accumulation and simultaneously enhances the content of anthocyanin and ascorbic acid (AsA). Overall, this study provides novel insights into the regulatory mechanisms of GABA accumulation in strawberry fruit and identifies *FaGAD4* and potential TFs as valuable genetic targets for molecular breeding.

## 1. Introduction

Strawberry (*Fragaria × ananassa*) is one of the most important horticultural crops, contributing to economic development and human health. Correspondingly, the unique flavor metabolites and nutrient compounds determine the fruit quality and commercial value. Generally, soluble sugars, organic acids, and volatiles determine most of the taste quality of strawberry fruit; in addition, phytochemicals such as AsA, flavonoids, organic acids, and GABA contribute significantly to the fruit’s antioxidant capacity and potential health benefits, including anti-inflammatory and cardioprotective effects [1,2,3,4]. The evolution of consumer preferences reflects a shift from a primary focus on taste towards a greater emphasis on the nutritional and health attributes of fruits [5]. Therefore, clarifying the health-promoting chemicals in strawberry fruits and illustrating the molecular mechanisms regulating the compounds’ homeostasis will provide insights for molecular breeding of strawberry cultivars to meet market demands.

GABA, a four-carbon non-proteinogenic amino acid, widely exists in plants, animals, and bacteria. In humans, GABA functions as an inhibitory neurotransmitter in the central nervous system [6], which has been reported to reduce blood pressure, alleviate anxiety, enhance immunity, and improve liver function. In plants, GABA is involved in plant growth, development, defense response, and carbon and nitrogen metabolism [7] and plays a crucial role in improving the antioxidant defense system of plants [8]. Recently, some studies have reported the role of GABA in improving fruit quality and extending the shelf life [9,10,11,12,13].

In higher plants, GABA is primarily metabolized via the GABA shunt pathway. Firstly, GABA is irreversibly synthesized from glutamate catalyzed by the key rate-limiting enzyme glutamate decarboxylase (GAD) and subsequently reversibly converted to succinic semialdehyde (SSA) by GABA transaminase (GABA-T). Succinate semialdehyde dehydrogenase (SSADH) further oxidizes the SSA to succinate, which is an important compound of the tricarboxylic acid (TCA) cycle [14,15,16]. In addition, degradation from putrescine and polyamines has also been reported and is considered an alternative pathway for GABA accumulation [17,18]. *GAD* is encoded by a small gene family in plants and post-translationally regulated by Ca^2+^/CaM, thereby maintaining appropriate GABA levels [19]. Five members are present in the *Arabidopsis thaliana* and tomato genome [16,20]. Overexpression of *SlGAD2* and *SlGAD3* significantly increased the GABA levels of tomato fruit [21]. Nowadays, further investigations remain to be conducted for the identification of *GAD* family members, along with functional analysis and clarification of the related molecular mechanisms in strawberry.

GABA is biosynthesized from glutamate and closely related to the TCA cycle in plants [22]. The TCA cycle is commonly thought to be responsible for the production of energy and offers carbon skeletons for anabolic processes [23]. The components of the TCA cycle, particularly citric acid and malic acid, contribute to both human health and flavor formation of strawberry fruits. Generally, they are crucial for the sugar–acid balance, constructing the complex sensory profile and enhancing the overall freshness and palatability of the fruit, and citric acid accounts for more than 50% of the total organic acid content in strawberry fruit [24,25]. Researchers have indicated that malate-associated QTLs are located in LGII-5 and LGIII-4, while citrate-associated QTLs are mapped to LG1-1 and LGV-2 in strawberry [26], and transcription factor *FaMYB73 is* involved in malic acid accumulation [27]; moreover, *FaGAPC2/FaPKc2.2* and *FaPEPCK* perform important roles in reducing citric acid content in strawberry fruits [28]. However, current knowledge about the accumulation patterns of organic acids in strawberry fruits and their underlying regulatory mechanisms remains limited.

The aim of this study was to elucidate the physiological and molecular mechanisms underlying the differential accumulation of GABA and organic acids in strawberry fruits by combining transcriptome analysis with functional characterization of key genes and to screen for potential regulatory transcription factors. We compared GABA and organic acid accumulation in two strawberry cultivars, ‘Monterey’ and ‘Benihoppe’. Through transcriptome analysis, we identified *FaGAD4* as a key gene not only for GABA biosynthesis but also for anthocyanin and AsA production. Furthermore, we uncovered the transcription factors involved in GABA accumulation, providing new genetic targets for enhancing strawberry nutritional quality.

## 2. Materials and Methods

### 2.1. Plant Materials

**Figure 1:**
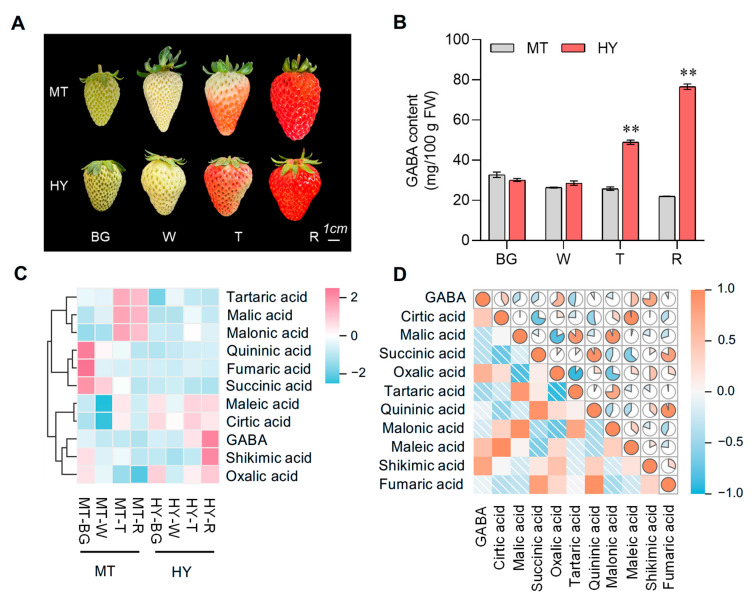
Phenotype observation and physiological analysis. (**A**) Four developmental stages of ‘Monterey’ and ‘Benihoppe’. Big green fruit stage (BG), white fruit stage (W), turning stage (T), red stage (R), ‘Monterey’ (MT), and ‘Benihoppe’ (HY). Scale bar: 1 cm. (**B**) GABA content in four fruit developmental stages of MT and HY. Statistical significance was determined by Student’s *t* test (** *p* < 0.01). (**C**) Cluster heatmap of GABA and organic acid content in four fruit developmental stages of MT and HY. (**D**) Correlation heatmap of GABA and organic acid content in four fruit developmental stages of MT and HY. The color scales 0–1 represent the Pearson correlation coefficient.

The octoploid strawberry (*Fragaria × ananassa*) cultivars ‘Monterey’ and ‘Benihoppe’ and diploid strawberry (*Fragaria vesca*) ‘Ruegen’ were cultivated in the sunlight greenhouse at the Institute of Forestry and Pomology, Beijing Academy of Agriculture and Forestry Sciences, Beijing, China. For octoploid strawberry, fruits at four developmental stages were collected, including big green (BG), white (W), turning (T), and red (R) [29] (Figure 1A). The fruit samples were rapidly frozen in liquid nitrogen and then stored at −80 °C for GABA and organic acid content determination as well as RNA sequencing (RNA-seq) analysis. Additionally, the fruits of ‘Ruegen’ were used for the transient transformation experiments. Three biological replicates were applied, and each repeat consisted of 10 fruits with uniform developmental stages in all of the fruit experiments.

### 2.2. Determination of GABA, Anthocyanin, and AsA Content

An Agilent liquid chromatograph equipped with a VWD detector (UltiMate 3000, Thermo Fisher Scientific, Waltham, MA, USA/HPLC 1200, Agilent Tec, Santa Clara, CA, USA) was used to profile the GABA content of strawberry fruits. Samples at four developmental stages of the two cultivars were mixed separately and ground to power with liquid nitrogen. Briefly, 0.2 g of pulverized samples was suspended with 5 mL of 6 mol/L hydrochloric acid (HCl) solution and vortex vigorously for 1 min. This was then hydrolyzed in 110 °C for 24 h. The hydrolyzed samples were retrieved and 5 mL of 6 mol/L sodium hydroxide solution was added before vortexing for 1 min. Centrifugation was performed at 5000 rpm for 10 min (HR2-16K, Hunan Kecheng Instrument Equipment Co., Ltd., Changsha, China). In total, 0.5 mL of supernatant was transferred to a 5 mL tube. Sequentially, 0.5 mL of 0.5 mol/L sodium bicarbonate buffer (pH 9.0) and 0.5 mL of 2,4-dinitrofluorobenzene (DNFB) solution were added. This was then incubated in a 60 °C water bath with dark conditions for exactly 60 min. The solution was diluted to 5 mL with pH 7.0 phosphate buffer and vortexed for 1 min, with dark treatment for 15 min. The 1 mL filtered supernatant was injected into the HPLC system for GABA determination [30]. The HPLC separation was performed on a Hypersil GOLD guard column (250 mm × 4.6 mm; 5 μm) at 25 °C. The solvents for the mobile phase were A (1 mol/L sodium acetate solution, pH 5.3) and B (methanol/water = 1:1 (*v*:*v*)). The flow rate was 1 mL/min and the injection volume was 20 μL. And the detection wavelength was 360 nm. Anthocyanin and AsA content were determined using commercially available kits (Beijing Boxbio Science & Technology Co., Ltd., Beijing, China) according to manufacturer’s instructions.

### 2.3. Determination of Organic Acid Content

HPLC was used for determining the content of organic acids (UltiMate 3000, Thermo Fisher Scientific, Waltham, MA, USA/HPLC 1200, Agilent Tec, Santa Clara, CA, USA) [31]. Strawberry fruits were pulverized in liquid nitrogen, 1 g of the samples was suspended in 4 mL pre-chilled deionized water and homogenized at low temperature for 200 s, and the samples were placed in a 4 °C water bath for ultrasonic oscillation extraction for 1 h. After that, the samples were kept at 4 °C for cold immersion extraction overnight and centrifuged for 10 min at 10,000 rpm, and the supernatant was collected into another 10 mL centrifuge tube. The entire extract was passed through an organic acid-specific purification column and eluted with 3 mL of 0.1 mol/L hydrochloric acid aqueous solution. After that, the eluate was passed through a 0.45 μm filter membrane for instrumental analysis. The HPLC separation was performed on a TSKgel ODS-100V guard column (4.6 mm × 250 mm × 5.0 μm, Tosoh, Shunan, Yamaguchi, Japan) at 25 °C. The solvents for the mobile phase consisted of a solution of KH_2_PO_4_ with a concentration of 0.02 mol/L and pH 2.4. The flow rate was 1 mL/min and the injection volume was 30 μL. The organic acid concentration was calculated by measuring UV absorbance at a wavelength of 210 nm. The detailed standard curve equations for all organic acids are provided in Supplementary Table S1.

### 2.4. RNA-Seq and Transcriptome Analysis

Total RNA of strawberry fruits was extracted using an E.Z.N.A.^®^ Total RNA Kit (Omega R6827-01, Norcross, GA, USA). The cDNA libraries were constructed and sequenced on an Illumina Genome Analyzer at Biomarker Technologies Corporation, Beijing, China. Clean reads were then mapped to the strawberry reference genome (*Fragaria × ananassa* camarosa v1.0) [32]. Adjusted *p*-value < 0.05 and |log2(fold change)| ≥ 1 were set as thresholds for screening significant differentially expressed genes (DEGs). The raw data of the RNA-seq has been submitted to the NCBI database (accession number PRJNA1345204).

### 2.5. WGCNA

To identify key gene modules significantly related to GABA and organic acid accumulation in strawberry fruits, we performed a weighted gene co-expression network analysis (WGCNA) package in R. The parameters were set as follows: soft threshold power: 7; minModuleSize: 50; and cutHeight: 0.25 [33]. Then the co-expression regulatory networks were visualized via Cytoscape (v3.8.1) [34].

### 2.6. Subcellular Localization of FaGAD4

The full-length coding sequence (CDS) without a stop codon of *FaGAD4* was amplified and inserted into the pRR002 vector. Recombinant and empty vectors were transformed into the GV3101 *Agrobacterium tumefaciens* strain. Then the 4- to 6-week-old tobacco (*Nicotiana benthamiana*) leaves were infiltrated with the *Agrobacterium* cells and incubated at 23 °C under an 8 h/16 h light/dark cycle for 72 h. Fluorescence signals were detected using a confocal laser scanning microscope (Nikon A1, Tokyo, Japan). Primers used in vector construction are listed in Supplementary Table S2.

### 2.7. Transient Expression of FaGAD4 in Strawberry Fruits

The CDS of *FaGAD4* was cloned into the pRR002 vector to transiently overexpress *FaGAD4* (*FaGAD4*-OE). *FaGAD4*-OE and empty vectors (OE and EV) were introduced into the *Agrobacterium tumefaciens* strain GV3101 individually and cultured at 28 °C in LB liquid medium. At an OD_600_ of 0.8, the agrobacterium cultures were centrifuged and resuspended in buffer (10 mM MES, pH 5.6, 10 mM MgCl_2_, and 100 µM acetosyringone) and activated at room temperature for 2 h. Subsequently, the buffer with agrobacterium was injected in the diploid strawberry ‘Ruegen’ fruits at the 15 DAA (days after anthesis) stage. EV and OE fruits were collected at 7 d after infiltration to analyze the GABA, anthocyanin, and AsA content, as well as the expression of *FaGAD4*. Three biological repeats were performed and each repeat consisted of 10 fruit individuals [35].

### 2.8. qRT-PCR Assays

The total RNA was isolated from the strawberry fruits with the E.Z.N.A.^®^ Total RNA Kit (Omega, Norcross, GA, USA). The cDNA was synthesized from 1 μg total RNA with EasyScript^®^ First-Strand cDNA Synthesis SuperMix (Transgene, Beijing, China). Then the qRT-PCR was carried out with TB GreenTM Premix Ex TaqTM II (Takara, Kyoto, Japan) via a CFX96TM Real-Time System (Bio-Rad, Hercules, CA, USA). The relative expression level was calculated using the 2^−ΔΔCt^ method corresponding to a housekeeper gene of *FvACTIN* [36]. The primers used in qRT-PCR analysis are shown in Supplemental Table S3.

### 2.9. Statistical Analysis

Statistical analysis was performed with GraphPad Prism (version 8.0), and statistically significant differences between samples were calculated using Two-tailed Student’s *t*-test (* *p* < 0.05, ** *p* < 0.01).

## 3. Results

### 3.1. Phenotype and Physiological Characteristics During Strawberry Fruit Ripening

The phenotype of ‘Monterey’ (MT) and ‘Benihoppe’ (HY) fruits at four different developmental stages are presented in Figure 1A. The GABA content slightly decreased during the fruit development and ripening in MT fruits, reaching approximately 20 mg/100 g fresh weight (FW) at the maturation R stage. In contrast, the GABA content gradually increased during fruit ripening, peaking at the R stage with a content of about 80 mg/100 g FW in HY fruits. At the green (G) and white (W) stages, there were no significant differences in GABA content between the two varieties. However, from the onset of fruit coloring, the GABA content in HY fruits was approximately twice that of MT (Figure 1B).

Organic acids are the primary source of acidity in strawberry fruits. Citric acid and malic acid are key intermediates of the tricarboxylic acid (TCA) cycle, which is tightly associated with the GABA shunt. In the present study, we determined the content of ten kinds of organic acids across fruit developmental and ripening in MT and HY (Supplementary Figure S1).

Cluster analysis revealed that the organic acids could be classified into two distinct groups in accordance with contents. GABA was clustered with maleic acid, citric acid, shikimic acid, and oxalic acid, and the contents of these compounds were higher in HY fruits and increased gradually during fruit ripening. On the contrary, tartaric acid, malic acid, malonic acid, quininic acid, fumaric acid, and succinic acid were more abundant in MT fruits. Among these, the contents of tartaric acid, malic acid, and malonic acid increased during fruit ripening in MT, while the levels of quininic acid, fumaric acid, and succinic acid presented an opposite trend (Figure 1C). Heatmap of correlations among the multiple metabolites indicated that GABA was significantly positively correlated with shikimic acid, oxalic acid, citric acid, and maleic acid (Figure 1D). The results comprehensively elucidate the differential accumulation patterns of GABA and organic acids between MT and HY fruits, providing physiological evidence for the distinct flavor and nutritional quality that appeared between varieties.

### 3.2. Transcriptome Analysis of MT and HY Fruits

**Figure 2:**
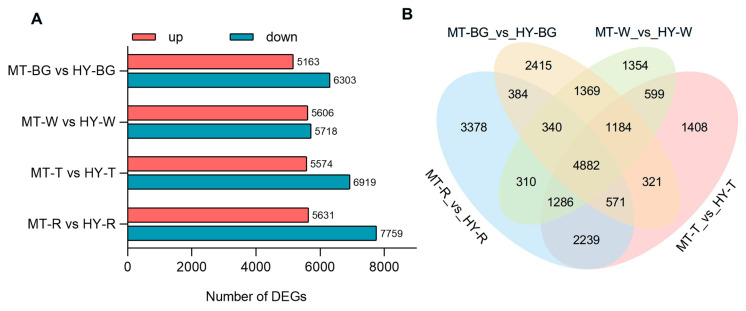
Overview of transcriptome. (**A**) Number of up- and down-regulated genes in two strawberry varieties with 4 pairwise comparisons. (**B**) Venn diagram of differently expressed genes (DEGs) for different pairwise comparisons.

To elucidate the molecular mechanisms behind the significant differences in GABA and organic acid accumulation between MT and HY, we performed transcriptome analysis at four fruit developmental stages. Based on the DEGs analysis, the largest divergence of DEGs occurred at the R stages, with 5631 genes up-regulated and 7759 genes down-regulated in HY fruits compared to MT (Figure 2A). Venn diagram analysis of the four differential comparison groups revealed that the MT-T_vs_HY-T and MT-R_vs_HY-R groups showed a relatively large number of DEGs (Figure 2B). GO and KEGG analysis illustrated the significant differences in the transcript levels of genes related to biosynthesis of amino acids and carbon metabolism (Supplementary Figures S2 and S3). Consequently, both the T and R fruit stages play key roles in fruit quality formation between cultivars. The DEGs identified at these crucial stages are prime candidates for functional studies underlying the molecular mechanisms involved in GABA and organic acid accumulation.

### 3.3. Establishment of ‘Metabolites vs. Gene’ Modules in Strawberry Fruit

**Figure 3:**
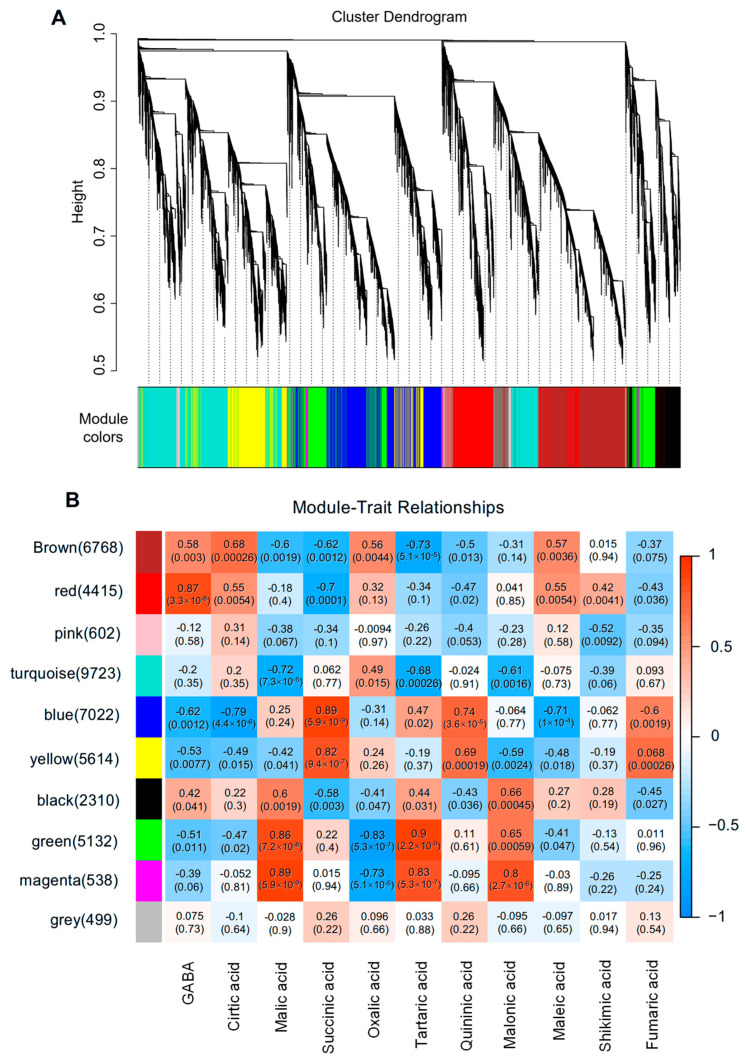
Correlation analysis of transcriptomic and metabolite contents during four fruit developmental stages in two strawberry cultivars. (**A**) Dendrogram indicating co-expression modules analyzed by WGCNA during four fruit developmental stages. (**B**) Heatmap showing correlations between gene modules and metabolites. Each column in a different color represents a gene module. Every row represents a metabolite. Red color and blue color indicate a positive and negative correlation between the gene clusters and the metabolites, respectively.

To gain further insights into the molecular mechanisms regulating the accumulation of GABA and organic acids throughout strawberry fruit development and ripening, weighted gene co-expression network analysis (WGCNA) was performed to clarify the co-expression networks of DEGs. A total of 10 distinct modules were identified based on their expression patterns (Figure 3A). Among these gene modules, the turquoise module contained the largest number of genes (9723), while the gray module contained the fewest genes (499). The red module comprised 4415 genes and showed a significantly positive correlation with GABA content. Both the brown module (6768) and the red module were closely related to citric acid content, whereas the green (5132) and magenta (538) modules exhibited a positive association with malic acid content (Figure 3B). These results indicate that genes within these four modules are mainly involved in GABA accumulation and acidity formation during strawberry fruit ripening.

### 3.4. Identification of Key Structural Genes for GABA Shunt and TCA Cycle

**Figure 4:**
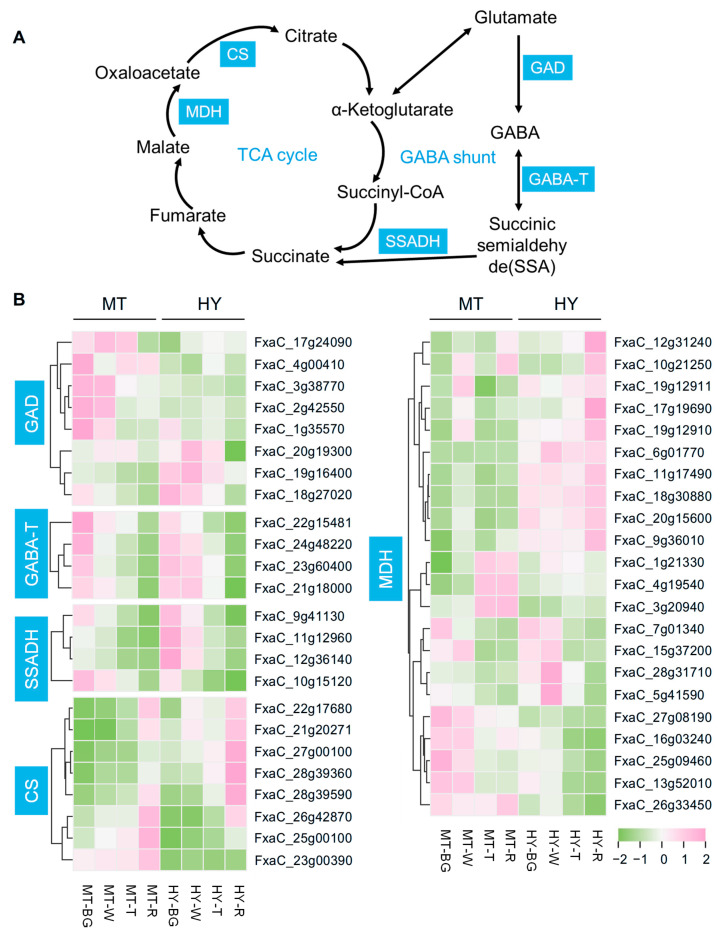
Identification of key structural genes in strawberry GABA, citric acid, and malic acid accumulation. (**A**) Metabolic pathways of the GABA shunt and TCA cycle. GAD, glutamate decarboxylase; GABA-T, GABA transaminase; SSADH, succinate semialdehyde dehydrogenase; CS, citrate synthase; MDH, malate dehydrogenase. (**B**) Heatmap showing the transcript levels of key structural genes in the GABA shunt and TCA cycle.

The biosynthesis and metabolism of GABA occur primarily via the GABA shunt [15]. This pathway is mediated by three key enzymes: GAD, GABA-T, and SSADH. Specifically, SSADH catalyzes the oxidation of succinic semialdehyde (SSA) to succinate, which subsequently serves as an important metabolite in the TCA cycle (Figure 4A). Transcriptomic analysis of MT and HY fruits identified key structural genes involved in the GABA shunt and TCA cycle. Within the TCA cycle, we focused on key candidates associated with the accumulation of citric acid and malic acid. Overall, 46 members were identified, including 8 *GAD* genes, 4 *GABA-T* genes, 4 *SSADH* genes, 8 *CS* genes, and 22 *MDH* genes. GAD functions as the rate-limiting enzyme for the GABA shunt. Among the eight *GAD* genes, five members exhibited higher expression levels in MT fruits compared to HY, while three members (FxaC_20g19300, FxaC_19g16400, and FxaC_18g27020) showed higher expression in HY fruits, suggesting that these three members may play critical roles in GABA accumulation during strawberry fruit ripening. However, the transcript levels of *GABA-T* members did not differ significantly between MT and HY fruits. The expression of *SSADH* was relatively higher in HY but exhibited a declining trend in both cultivars, which was opposite to the accumulation pattern of GABA in HY fruits (Figure 1B). CS and MDH are key enzymes in the TCA cycle for citrate and malate accumulation, respectively. The five *CS* candidates showed higher expression in HY, with an increasing trend during fruit ripening, and the other three members were more highly expressed in MT. According to the cluster heatmap, the transcript abundances of ten *MDH* members were higher in HY fruits, and among the remaining twelve members, eight presented higher expression levels in MT, while the other four exhibited no significant differences between MT and HY, with overall downward trends (Figure 4B).

### 3.5. Identification of Key Transcription Factors Regulating GABA Accumulation

**Figure 5:**
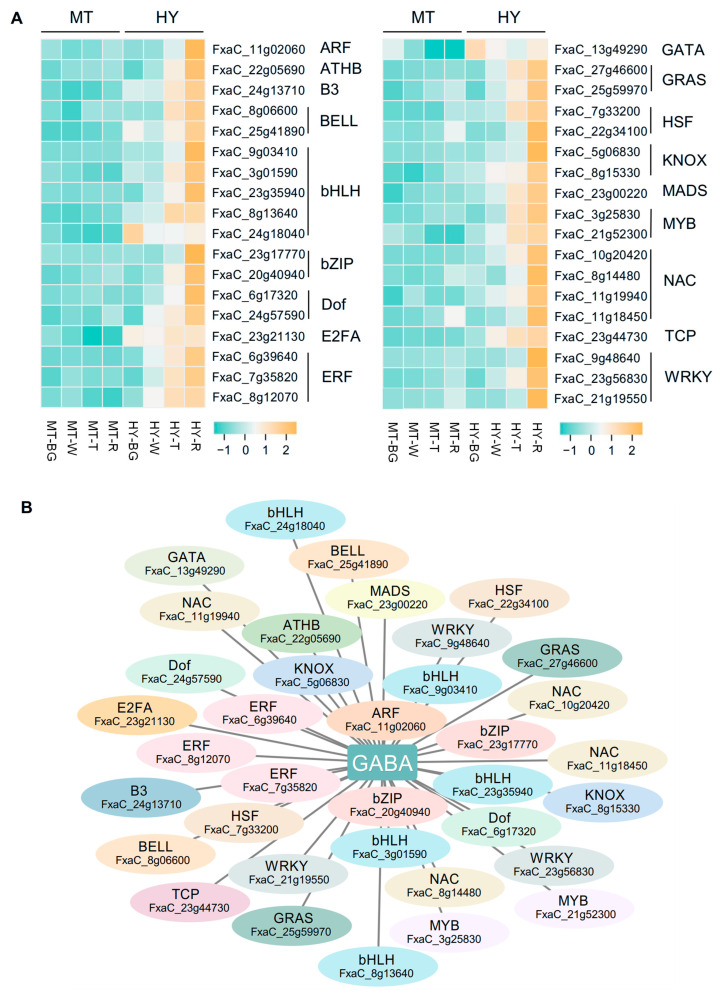
Identification of potential transcription factors in strawberry GABA biosynthesis. (**A**) Heatmap showing the expression patterns of candidate transcription factors across four fruit stages in MT and HY. (**B**) Network showing the correlation of GABA and TFs.

To identify key transcription factors (TFs) regulating GABA accumulation, we analyzed genes within the “red” module obtained from WGCNA (Figure 2B), which showed a significantly positive correlation with GABA content. A total of 36 TFs belonging to 18 families were screened and exhibited higher expression in HY fruits, with increased trends during fruit development and ripening, whereas their transcript levels were lower and remained stable during fruit ripening in MT fruits (Figure 5A). The co-expression network depicted in Figure 5B illustrates the degree of significance by positional relationship. Genes located closer to GABA in the network were more strongly associated with its accumulation. Further molecular biological approaches will be applied to characterize the regulatory roles of these candidate TFs in GABA accumulation in strawberry fruits (Figure 5B).

### 3.6. FaGAD4 Promotes GABA, Anthocyanin, and AsA Accumulation in Strawberry Fruits

**Figure 6:**
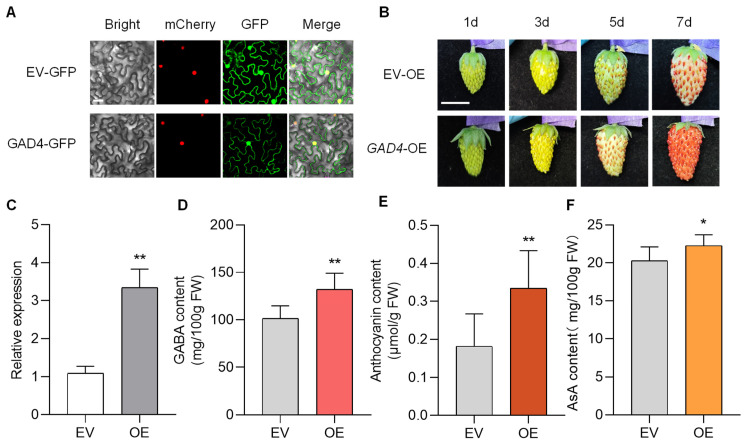
*FaGAD4* promotes GABA, anthocyanin, and AsA accumulation in strawberry fruits. (**A**) Subcellular localization of FaGAD4, scale bar: 20 μm. (**B**) Phenotypes of EV and OE fruits: EV, empty vector; OE, *FaGAD4* overexpression. Scale bar: 1 cm. (**C**) The expression levels of *FaGAD4* in EV and OE fruits. (**D**) GABA content in EV and OE fruits. (**E**) Anthocyanin content in EV and OE fruits. (**F**) AsA content in EV and OE fruits. Statistically significant differences from the control were determined by Student’s *t*-test: * *p* < 0.05; ** *p* < 0.01.

The role of the *FaGAD* genes in GABA accumulation in strawberry remains to be elucidated. In this study, eight GAD members were identified through transcriptomic analysis, and their expression levels were verified by RT-qPCR (Figure 4B, Supplementary Figure S5). Among these, three GAD members showed higher transcript levels in the high-GABA variety HY. Among them, the expression of FxaC_19g16400 was consistently higher in HY across all four fruit developmental stages compared with MT, particularly at the T and R stages. The differential expression of FxaC_19g16400 between varieties was significantly greater than that of FxaC_20g19300 and FxaC_18g27020 (Supplementary Figures S4 and S5). Therefore, we speculated that FxaC_19g16400 plays a dominant role in regulating GABA accumulation and named it *FaGAD4* based on the transcriptome annotation. Subsequently, we investigated the function of *FaGAD4* in GABA accumulation and quality formation in strawberry fruit. Subcellular localization analysis revealed that the FaGAD4 protein was localized in the cytoplasm and nucleus (Figure 6A). Additionally, we transiently overexpressed *FaGAD4* in diploid strawberry fruits (Figure 6B,C). At 7 days after transformation, *FaGAD4* overexpression significantly enhanced the accumulation of GABA in strawberry fruits (Figure 6D), leading to notably increased levels of anthocyanin (Figure 6B,E) and AsA (Figure 6F).

## 4. Discussion

Due to the increasing awareness of the health-promoting benefits, nutrient biofortification has emerged as a new target for crop breeding [20]. In this study, we selected two classic representative strawberry varieties: ‘Monterey’ (MT), a day-neutral variety from California, USA, characterized by high productivity and strong disease resistance, and ‘Benihoppe’ (HY), a short-day variety from Japan, famous for its excellent sweet–sour flavor and superior quality. Recently, a study revealed the distinctions and characteristics of strawberries with different fruit colors regarding texture, flavor, and color formation processes in ‘Benihoppe’ and ‘Fenyu No.1’ [37]. In the present study, we applied HPLC to systematically analyzed the contents of soluble sugars, organic acids, GABA, AsA, and plant hormones at four different fruit development stages in MT and HY (some data have not been provided in this paper). Specifically, we focused on GABA, a crucial nutrient, and organic acids, key metabolites of fruits that are poorly understood in strawberry.

### 4.1. Variation in GABA and Organic Acid Contents Between Two Strawberry Cultivars During Fruit Ripening

GABA has recently received considerable attention as a health-promoting functional compound, and it also serves as a non-protein amino acid or signaling molecule to improve plant growth and development, modulating the antioxidant system [38,39,40]. Furthermore, some studies have revealed that GABA plays a positive role in maintaining fruit quality in fruits such as grape, apple, peach, and citrus [9,10,11,12,41]. In strawberry, recent research has indicated that GABA contributes to improved fruit quality and extended shelf life by modulating the transcripts of genes related to citrate metabolism [13]. Our results indicated that the GABA content in ripening fruits of MT and HY strawberries was approximately 22 mg/100 g FW and 77 mg/100 g FW, respectively. Previous studies found that the strawberry cultivars ‘Allstar’ and ‘Earliglow’ had the lowest GABA concentrations at harvest, at 1.6 mg/100 g FW. The highest GABA levels were detected in ‘Jewel’ with an average of 3.6 mg/100 g FW, while those of ‘Northeaster’ were 2.5 mg/100 g FW [4]. It has been reported that GABA content in tomatoes varies significantly from 7 to 201 mg/100 g FW, depending considerably on genotype or cultivar [20,42]. In jujube, the GABA content was found to be 5.12–14.02 mg/100 g FW [43], while in litchi, it ranged from 60 to 130 mg/100 g FW [44], and longan fruit had high GABA content, ranging from 51.48 mg/100 g to 180.42 mg/100 g [45]. It is noteworthy that GABA content increases significantly or remains stable during strawberry fruit ripening, while in tomato fruits, GABA levels decrease markedly throughout the ripening process [41], reaching their lowest point in fully ripe fruits. This distinctly different accumulation pattern warrants further investigation. As for organic acids, a previous study revealed that the citric acid content ranged from 6.70 to 62.64 mg/g FW, while the malic acid content ranged from 0.19 to 10.29 mg/g FW in ripening strawberry fruits [25]. Our data indicated that citric acid content reached about 4.71 (‘Monterey’) and 5.82 (‘Benihoppe’) mg/g FW, whereas malic acid levels were 1.85 and 0.829 mg/g FW in mature fruits, respectively (Supplementary Figure S1).

### 4.2. FaGAD4 Positively Regulates GABA Accumulation in Strawberry Fruits

The *GAD* gene was recognized as the core regulatory node of the GABA shunt [46,47]; we identified that a total of eight *FaGAD* gene members were expressed in strawberry fruits, and among these, *FaGAD4* (FxaC_19g16400) present the most significant difference in transcript level between two cultivars fruits. The FaGAD4 protein was localized in both the cytoplasm and nucleus, whereas DlGAD3 in longan was found exclusively in the cytoplasm [45]. It is necessary to explore the mechanistic and functional difference in the distinct subcellular localization of GAD across different species. To further verify the function of *FaGAD4* in GABA accumulation, we transiently overexpressed *FaGAD4* in strawberry fruits and the results demonstrated that *FaGAD4* indeed promote GABA accumulation in strawberry fruits, as well as anthocyanin and AsA accumulation. Consistent with this knowledge, the specific regulatory mechanisms of *FaGAD4* in the above metabolites’ accumulation need to be further clarified. In tomato, overexpression of *SlGAD2/SlGAD3* increased the GABA levels in the fruit to 2.7–5.2 times those of the wild-type [21], and *SlGAD2* plays a dominant role in coordinating GABA-associated developmental and stress adaptation processes [48]. Mutations at the C-terminal region of *SlGAD2* and *SlGAD3* via CRISPR/Cas9 produce GABA-enhanced tomato fruits [49]. Sicilian Rouge tomatoes, which are genetically edited to contain high amounts of GABA, have been sold directly to consumers in Japan by Tokyo-based Sanatech Seed Co. Ltd, Tokyo, Japan. [50]. Future studies aimed at breeding GABA-enriched novel strawberry cultivars with higher efficiency and precision gene editing methods would be fascinating.

To further investigate the transcriptional regulatory mechanism of GABA accumulation, we screened potential TFs based on the DEGs and differentially accumulated metabolites. To date, there are no reports on the transcriptional regulation of GABA accumulation in strawberry fruits. In apple and tomato, *GAD* was positively regulated by MdCBF3 [51] and SlNLP7 [52] and negatively regulated by MdNAC104 [53] and SlTHM27 [54]. Notably, among the TFs we identified, several families, including ATHB, B3, BELL, E2FA, KNOX, have not been functionally characterized in strawberry. It would be valuable to explore these TFs via molecular biology techniques to broaden our understanding of transcriptional regulatory networks in GABA accumulation and fruit quality formation of strawberry.

### 4.3. GABA Metabolism Is Related to the TCA Cycle

The metabolism of GABA bypasses the TCA cycle, and GABA serves as an intermediate metabolite in primary C/N metabolism [55]. Citric acid and malic acid are the crucial compounds of the TCA cycle, which determine the strawberry fruit acidity and enhance fruit flavor. CS is a rate-limiting enzyme in the TCA cycle that promotes citric acid accumulation in fruits like kiwifruit [56] and strawberry [28]; additionally, MDH catalyzes the reversible reaction between oxaloacetate (OAA) and malic acid [57]. Previous studies revealed that the content of malic acid was significantly increased in *MdMDH5*-overexpressed apple [58]. In this study, compared with MT fruits, the transcription levels of five *CS* genes and ten *MDH* members were up-regulated in HY fruits. Consequently, we speculated that those candidates play positive roles in citric acid biosynthesis and malic acid degradation, contributing to elevated citric acid levels and reduced malic acid levels in HY fruits. Interestingly, the succinic acid (succinate) content was approximately the same between MT and HY ripening fruits; thus we proposed a hypothesis that on one hand, HY fruits contain more glutamate as a substrate to biosynthesize more GABA through the GABA shunt, and on the other hand, GABA may be produced via oxidative stress-induced breakdown of polyamines, ultimately resulting in a higher accumulation of GABA in HY fruits [16].

To sum up, further investigations are essential to characterize the candidate TFs and structural genes identified in this study and to elucidate the mechanisms regulating GABA and organic acid accumulation. Additionally, future research should aim to clarify the molecular network of *GAD* genes in modulating GABA accumulation and other quality traits, such as anthocyanin and AsA, thereby facilitating GABA biofortification and promoting strawberry fruit quality through molecular breeding.

## 5. Conclusions

In summary, this study quantified GABA and organic acid content in two representative strawberry varieties across four fruit developmental stages and investigated the transcript levels of *GAD*, GABA-T, SSADH, *CS* and *MDH* in ‘Monterey’ and ‘Benihoppe’ strawberry fruits. Furthermore, 36 transcription factors from 18 distinct families were identified as potentially involved in the regulation of GABA accumulation in strawberry fruit. Functional validation revealed that *FaGAD4* plays a key role in positively regulating GABA accumulation, while also enhancing anthocyanin and AsA content, thereby improving the fruit’s antioxidant capacity. This study preliminarily establishes a regulatory network for GABA accumulation in strawberry, providing a theoretical foundation for understanding the metabolic regulation of GABA, citric acid, and malic acid. It also offers genetic resources for developing new strawberry varieties with improved nutritional and sensory qualities to meet consumer preferences.

## Data Availability

The data presented in this study are available in the article and Supplementary Material.

## Supplementary Materials

The following supporting information can be downloaded at https://www.mdpi.com/article/10.3390/plants14223437/s1, Figure S1: Contents of organic acids in four fruit developmental stages of ‘Monterey’ (MT) and ‘Benihoppe’ (HY); Figure S2: GO pathway annotation of differentially expressed genes at four stages in two strawberry varieties. ‘Monterey’ (MT) and ‘Benihoppe’ (HY); Figure S3: KEGG pathway annotation of differentially expressed genes at four stages in two strawberry varieties. ‘Monterey’ (MT) and ‘Benihoppe’ (HY); Figure S4: Transcript levels of three *GADs* in ‘Monterey’ (MT) and ‘Benihoppe’ (HY) fruits; Figure S5: Relative expression of eight *GADs* in ‘Monterey’ (MT) and ‘Benihoppe’ (HY) fruits; Table S1: Standard curves of 10 organic acids; Table S2: Primers used in vector construction; Table S3: Primers used in qRT-PCR analysis.
