# Supplementary material for: Identification of Key Regulators Mediating Gamma-Aminobutyric Acid (GABA) and Organic Acid Accumulation in Strawberry
Source: Plants (Basel). 2025 Nov 10;14(22):3437. doi: 10.3390/plants14223437 (PMC12655533; doi:10.3390/plants14223437)
Supplement: Supplementary file 1 [file plants-14-03437-s001.zip › Supplemental_Figures.pdf]

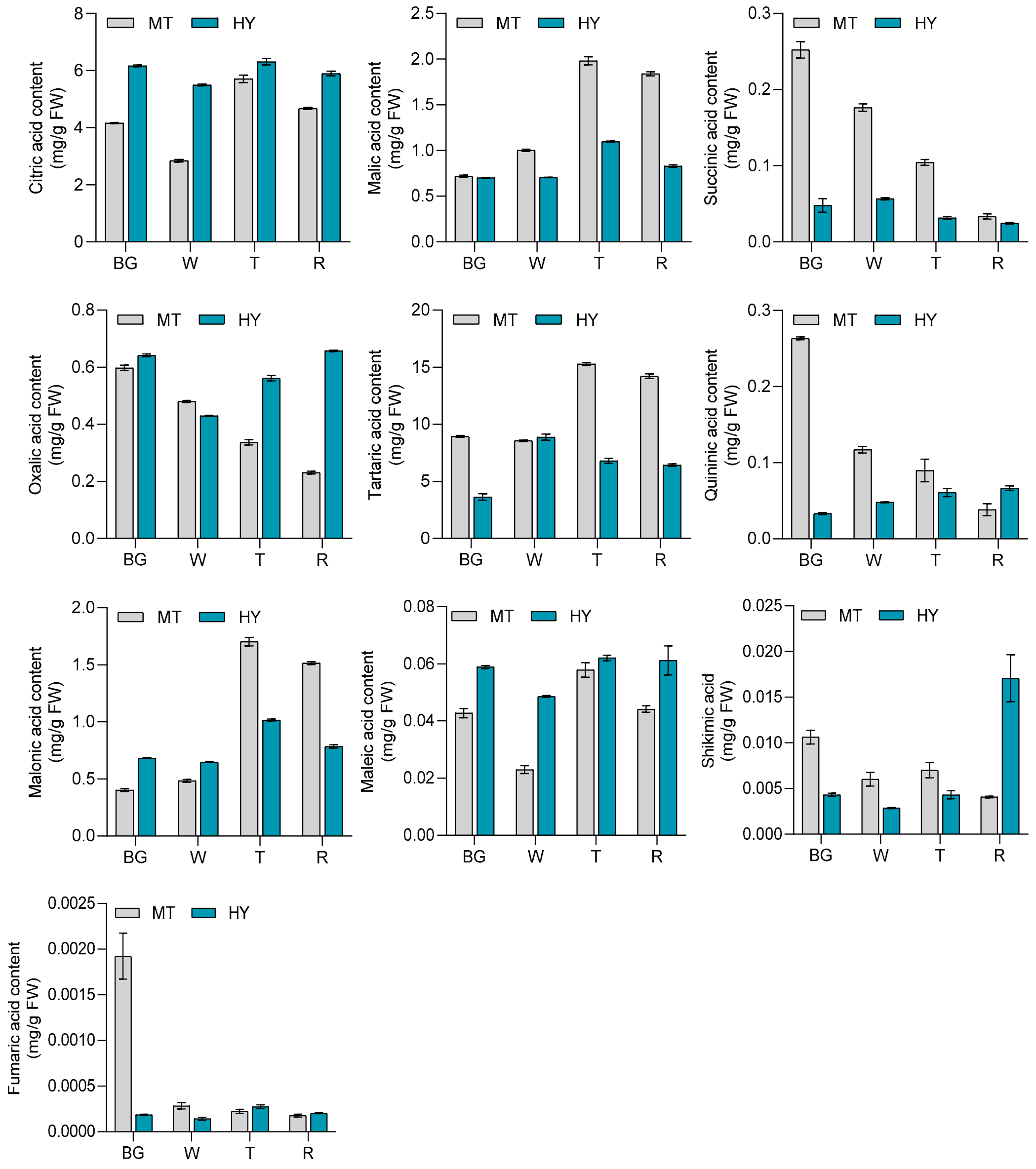

Supplemental Figure S1. Contents of organic acids in four fruit developmental stages of 'Monterey' (MT) and 'Benihoppe' (HY). Big green fruit stage (BG), white fruit stage (W), turning stage (T), red stage (R).

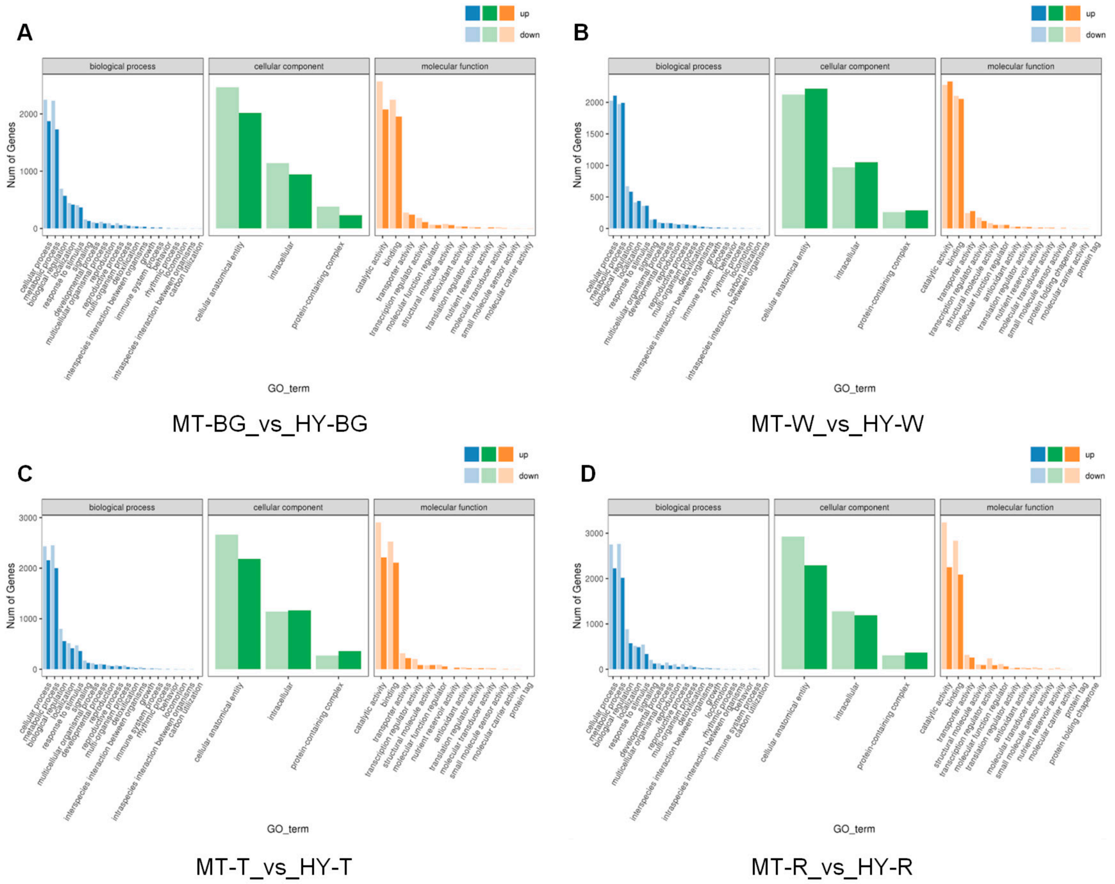

Supplemental Figure S2. GO pathway annotation of differentially expressed genes at four stages in two strawberry varieties. 'Monterey' (MT) and 'Benihoppe' (HY), Big green fruit stage (BG), white fruit stage (W), turning stage (T), red stage (R).

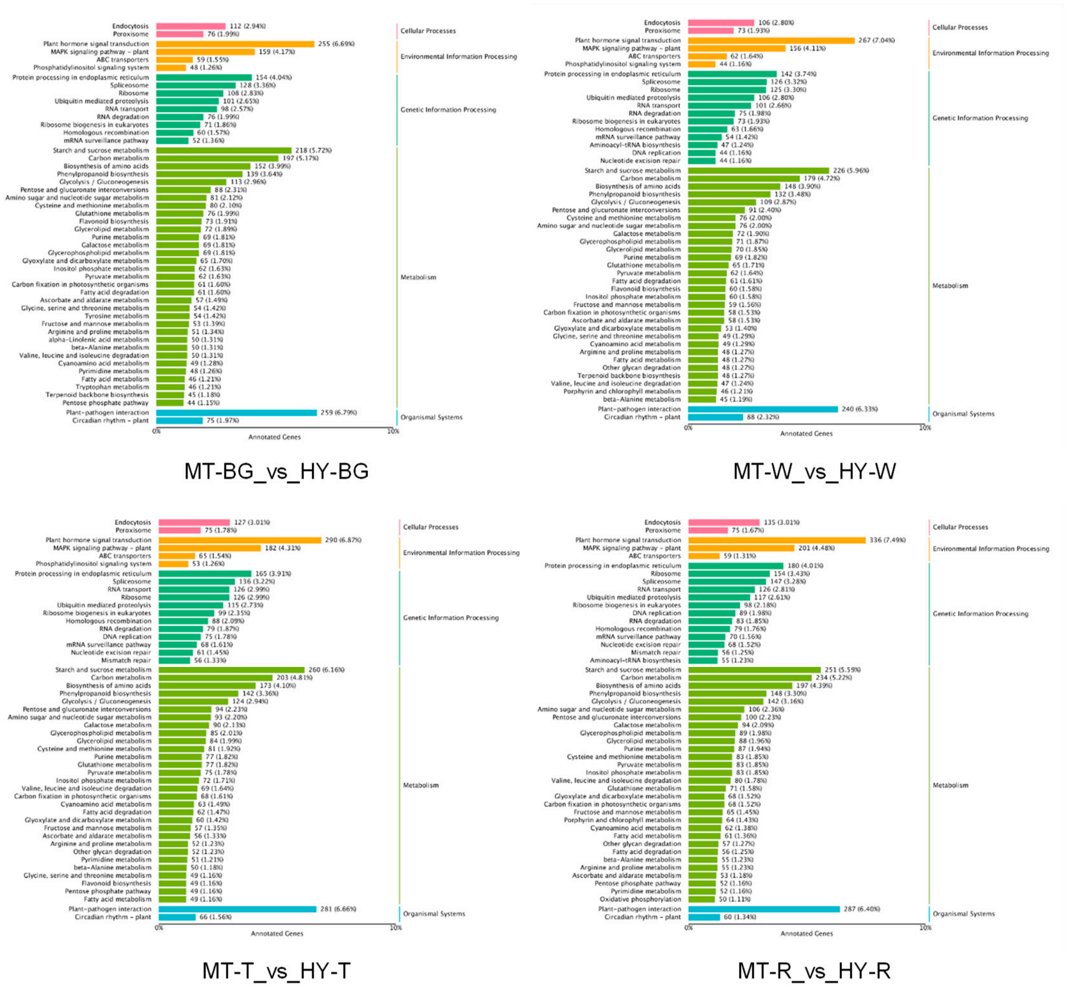

Supplemental Figure S3. KEGG pathway annotation of differentially expressed genes at four stages in two strawberry varieties. 'Monterey' (MT) and 'Benihoppe' (HY), Big green fruit stage (BG), white fruit stage (W), turning stage (T), red stage (R).

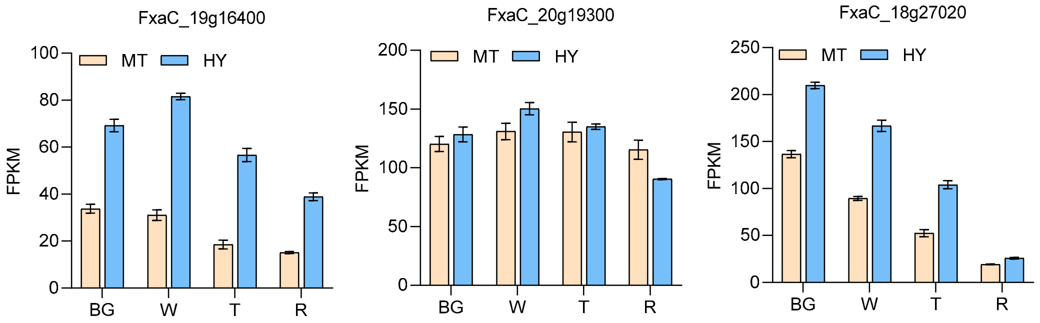

Supplemental Figure S4. Transcript levels of three *GADs* in 'Monterey' (MT) and 'Benihoppe' (HY) fruits. Big green fruit stage (BG), white fruit stage (W), turning stage (T), red stage (R).

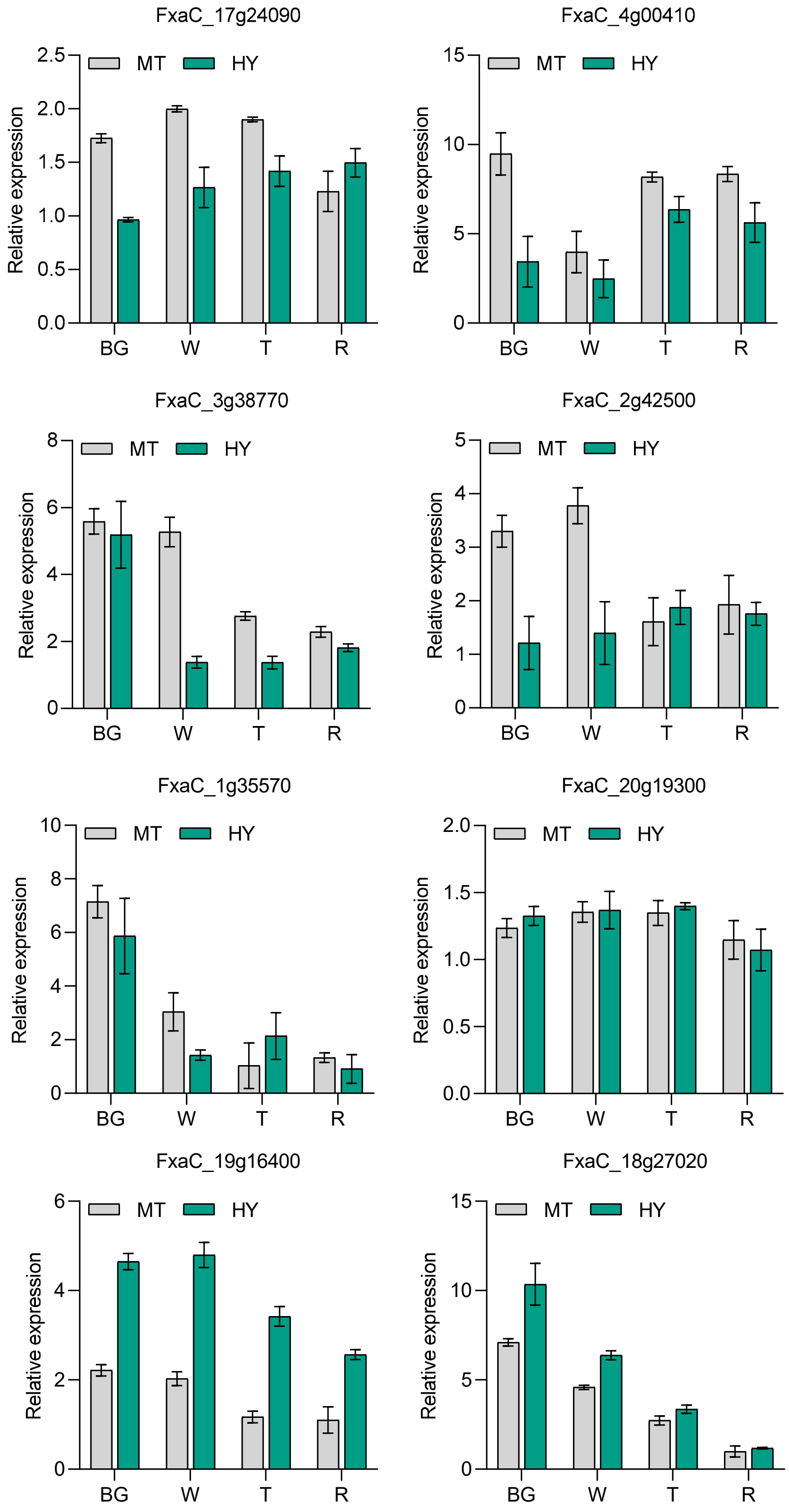

Supplemental Figure S5. Relative expression of eight *GADs* in 'Monterey' (MT) and 'Benihoppe' (HY) fruits. Big green fruit stage (BG), white fruit stage (W), turning stage (T), red stage (R).
